# Animal models and biomarkers of neuropathy in diabetic rodents

**DOI:** 10.4103/0253-7613.66833

**Published:** 2010-06

**Authors:** A.S. Shaikh, R.S. Somani

**Affiliations:** Department of Pharmacology, Sinhgad College of Pharmacy, Pune - 411 041, India

**Keywords:** Biomarker, diabetic neuropathy, diabetic rodents

## Abstract

Diabetic neuropathy (DN) is a multifactor complication of diabetes. It is a late finding in type 1 diabetes, but can be an early finding in type 2 diabetes. The cause of DN is still unclear and, like other complications of diabetes, it may be the result of various pathological conditions. Animal models and biomarkers of DN have been extensively used in neuropathic research. The most useful model of DN should exhibit the key feature present in human pathology. Diabetic rodents show behavioral, functional, structural and molecular biomarkers and they are widely used as models to investigate the etiology of DN as well as to screen the efficacy of the potential therapeutic interventions. We have reviewed the different animal models and biomarkers of neuropathy in diabetic rodents of either type 1 or type 2 diabetes.

Neuropathy is the most common complication of diabetes mellitus (DM). It occurs in 60% of the patients and affects their quality of life.[1] In the United States, diabetic neuropathy (DN) is the leading cause of diabetes-related hospital admissions and nontraumatic amputations.[2] In order to identify new treatments of DN, it is necessary to select the precise animal model. The selected animal model of DN should exhibit the features present in human pathology. Diabetic rodents show many abnormalities that are seen in diabetic patients with neuropathy, including hyperalgesia, allodynia, slow nerve conduction velocity (NCV) and progressive sensory and sensory motor deficit.

The decision to select the animal model for a particular protocol is very important. There are advantages and disadvantages to each model for the investigation of rodent DN. Contributing factors to the neuropathy phenotype in rodents include background strain, diet composition, insulin/C-peptide deficiency, coexisting hyperglycemia and hypertension and duration of diabetes. It is important to know about the different biomarkers of neuropathy in diabetic rodents and the time it takes to develop after the induction of diabetes.

The aim of this review is to highlight the available animal models and biomarkers of DN in rodents. This will provide adequate tools to investigate the mechanism of action of drugs with potential activity in DN as well as the etiological factors in the pathogenesis of DN.

## In Vivo Animal Models of DN

There are various models available, including models of type 1 and type 2 diabetes, for the study of neuropathy in rodents. Animal models that are used to study DN are broadly divided into two classes: induced models and genetic models. Induced models are further subdivided as drug-induced and diet-induced models of DN [Table 1].

**Table 1:** Neuropathic changes occurring in induced models of diabetes

*Animal models*	*Neuropathic changes*
Streptozotocin induced	Slow nerve conduction velocity, slow nerve blood flow, chemical, thermal and mechanical hyperalgesia, mechanical and chemical allodynia, increased sciatic nerve glucose, sorbitol and fructose, reduced nerve myoinositol, GSH, taurine concentration, lower (Na/K)-ATPas activity, delayed small intestinal transit, loss of epidermal nerve fiber, increased nerve water content, fasicular area and the area between myelineted fiber, axonal degeneration and Schewann cell proliferation
Alloxan induced	Slow nerve conduction velocity, thermal hyperalgesia, cold allodynia, delayed small intestinal and colonic transit, decreased gastric emptying, up-regulation of marker of oxidative stress
Galactose-enriched, diet induced	Slow nerve conduction velocity, increased nerve water content and fascicular area, accumulation of polyols, slight reduction of fiber diameter, reduced mean caliber of myelinated axons in both the saphenous and sciatic nerves
High-fat diet induced	Slow nerve conduction velocity, thermal hypoalgesia, tactile allodynia, up-regulation of markers of oxidative stress, increased sciatic nerve glucose, sorbitol and fructose

Streptozotocin (STZ) and alloxan (ALX) are two commonly used drugs to induce diabetes in animal models. A single dose of STZ can produce diabetes in rodents, probably as a result of its direct toxic effect on β-cell by alkylation, while ALX toxicates the β-cell by the generation of reactive oxygen species.

Early symptoms of neuropathy seen in STZ and ALX diabetic rodents include hyperalgesia, allodynia and slow NCV.[4–7] In addition to this, late symptoms of neuropathy such as hypoalgesia, motor incoordination, nerve degeneration, demylination and loss of epidermal nerve fiber are manifested in STZ diabetic rodents.[8,9]

The models for type 2 diabetes used in the studies of DN include genetic models such as BBZDR/Wor rat, Goto Kakizaki (GK) rat, Zucker diabetic fatty (ZDF) rat, Nagoya-Shibata-Yasuda (NSY) mouse, Otsuka Long-Evans Tokushima fatty rats (OLETF) and Db/db mouse.

In addition, there are nutritionally induced type 2 models such as the high-fat diet (HFD) C57Blk/6J mouse, galactose-enriched feed diet, in which the physiology can be studied. The HFD-fed mouse is a model for studying the pathogenesis of neuropathic changes developing in human subjects with IGT, obesity and metabolic syndrome.[10] The symptom of neuropathy manifested in the galactose-enriched feed diet includes slow NCV, increased nerve water content and fascicular area, accumulation of polyols, slight reduction of fiber diameter and reduced mean caliber of myelinated axons in both the saphenous and the sciatic nerves.[11,12] Similarly, slow NCV, thermal hypoalgesia, tactile allodynia, up-regulation of markers of oxidative stress and increased sciatic nerve glucose, sorbitol and fructose was seen in HFD-fed mouse.[13]

Because the prevalence of type 2 diabetes in humans is approximately 90%, these models have proven important in the studies of diabetic complications. In human type 2 diabetes, neuropathy often precedes the diagnosis of diabetes, making it difficult to stage these patients. It is hoped that animal studies of type 2 diabetes can lead to more meaningful details of the progression of nerve dysfunction in these patients. Less well studied are the genetic models of type 1 diabetes, such as non-obese diabetic (NOD), Biobreeding/Worchester (BB/Wor), Long Evans Tokushima lean rat (LETL), Chinese hamster LEW1AR1-iddm rat and Ins2C96Y Akita mouse. Various neuropathic changes manifested in genetic models of diabetes are mentioned in Table 2.

**Table 2:** Neuropathic changes occurring in the genetic models of diabetes[3]

*Animal models*	*Neuropathic changes*
Type I diabetes	
Non-obese diabetic (NOD)	Slowed nerve conduction velocity, thermal hypoalgesia, axonal degeneration on morphometry
Biobreeding/Worchester (BB/Wor)	Slowed nerve conduction velocity
Long Evans Tokushima lean rat (LETL)	Not well characterized in the literature
Chinese Hamster	Slowed nerve conduction velocity, loss of large myelinated fibers in proximal nerves, prominent autonomic neuropathy
LEW.1AR1-iddm rat	Not well characterized in the literature
Type II diabetes	
BBZDR/Wor rat	Slowed nerve conduction velocity, reduced Na/K+ ATPase activity in the nerves, myelinated fiber atrophy
Goto Kakizaki (GK) rat	Loss of small myelinated fibers, thermal hypoalgesia, reduced nerve conduction velocity
Zucker diabetic fatty (ZDF) rat	Decreased endoneurial blood flow, slowed nerve conduction velocity, elevated nerve sorbitol levels, thermal hyperalgesia
Nagoya-Shibata-Yasuda (NSY) mouse	Not well characterized in the literature
Otsuka Long-Evans Tokushima fatty rats (OLETF)	Slow nerve conduction velocity, reduced sciatic nerve blood flow, reduced Na/K+ ATPase activity in the nerves, decreased myelinated fiber size, abnormal autonomic activity
Db/db mouse	Paranodal axonal degeneration, slowed nerve conduction velocity, impaired axonal transport

## Biomarkers of Neuropathy in the Rodent Models of Diabetes

**Figure 1:**
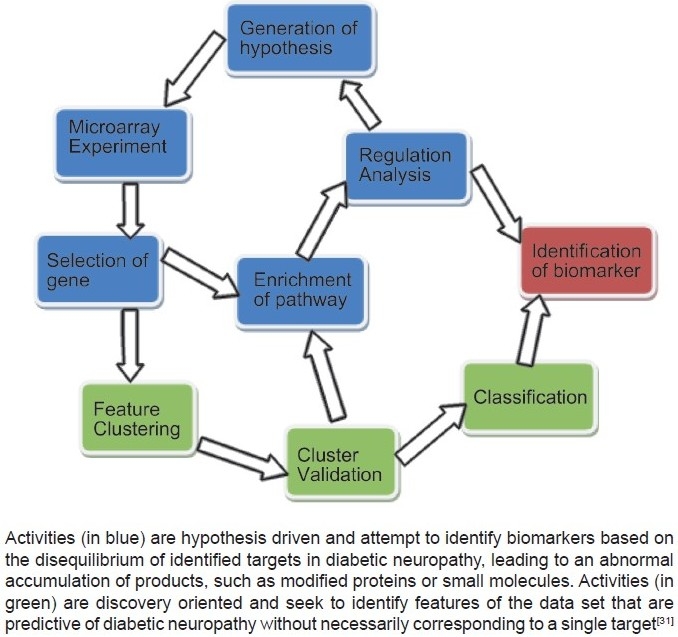
Methodologies of biomarker research in diabetic neuropathy[31] Activities (in blue) are hypothesis driven and attempt to identify biomarkers based on the disequilibrium of identified targets in diabetic neuropathy, leading to an abnormal accumulation of products, such as modified proteins or small molecules. Activities (in green) are discovery oriented and seek to identify features of the data set that are predictive of diabetic neuropathy without necessarily corresponding to a single target[31]

To identify the stage, progress of disease and new therapeutic interventions, a new, appropriate and widely utilizable biomarker should be identified, similar to microalbuminuria in the case of nephropathy that has proved to be a valuable means of detecting the stage of the disease. Such simple and easily available biomarkers of DN are currently lacking. Therefore, it is needed to identify such biomarkers of neuropathy in animal models of diabetes [Figure 1].

Some of these biomarkers of neuropathy reported in diabetic rodents may be functional, behavioral, structural and molecular.

## Behavioral biomarkers

Behavioral biomarkers include neuropathic pain, which is characterized by mechanical/chemical hyperalgesia, tactile allodynia in sensory large fibers, thermal nociception in sensory small fibers and sensory motor deficit in large sensory fibers.

**a) Neuropathic pain:** Neuropathic phenotyping in animal models begins with the evaluation of sensory loss by a quantitative assessment of neuropathic pain. The hallmark of this type of pain is allodynia or the elicitation of pain by a stimulus that is not normally considered noxious, such as light touch, pressure or mild temperature changes and hyperalgesia or pain sensation to less pain stimulation [Table 3].

**Table 3:** Biomarkers of neuropathic pain in diabetic rodents

Biomarkers of neuropathic pain	Reported time of development after induction of diabetes
Hyperalgesia	2 weeks after induction of diabetes, and remaining up to 6 weeks
Hypoalgesia	6 weeks
Allodynia	2 weeks after induction of diabetes, remaining up to 8 weeks

Neuropathic pain in diabetic rodents can be assessed by using thermal, mechanical and chemically induced pain,[8–10] which are enlisted in Table 4. There are several reports examining duration-dependent thermal, chemical, mechanical hyperalgesia and allodynia induced in experimental diabetic animal models. Short-term diabetes has shown thermal, chemical and mechanical hyperalgesia,[15–18] whereas long-term diabetes has shown thermal and mechanical hypoalgesia.[8] These duration-dependent changes in the thermal and mechanical nociceptive threshold reflect the symptoms in human DN. Thermal hyperalgesia is well observed in the early stage of DM in human subjects,[16] and longer term of DM shows an increased thermal threshold, which is caused by the loss of all types of peripheral nerve fibers. Thermal allodynia to warm stimulus of 42°C[18] and cold allodynia to cold stimulus of 10°C[7] were also observed from the second week of diabetes.

Heating the plantar surface of the paw to evoke withdrawal responses in diabetic rodents also show both hypoalgesia and hyperalgesia.[19–21] In STZ-diabetic rats, thermal hypoalgesia develops in the hind paw after approximately 8 weeks.[21]

**Table 4:** Tests for assessment of neuropathic pain in rodent models of diabetes

*Method*	*Test*
Thermal method	Tail immersion test Hot plate test
Mechanical method	Paw pressure test Tactile allodynia using van Frey hairs
Chemical method	Injection of a high concentration of formalin (0.5–5%) into the paw
	Injection of a low concentration of formalin (0.2%) into the paw

In addition to thermal allodynia, diabetic rats show a marked tactile allodynia when submitted to light touch to the plantar hind paw as well as chemical allodynia to paw injection of lower concentration of formalin (0.2%), while normal rats are insensitive to these stimuli. Tactile allodynia are notable within the first week of diabetes and remain up to 4 weeks, with worsening of the trouble from the second week onwards.[15] Allodynia in response to a 0.2% formalin stimulus is detectable after 1 week of hyperglycemia, and maximum worsening of symptoms occurs from 4 weeks to 8 weeks.[15]

**b) Motor incoordination:** The sensorimotor deficits resulting from large-fiber diabetic peripheral neuropathy (DPN) can lead to significant impairment. Numerous human studies report that patients with DPN are at an increased risk of falls due to decreased postural control and altered gait and balance.[22] Muscle spindles are involved in many sensorimotor behaviors such as the regulation of proprioception, balance, gait and the postural response, and spindle damage can lead to deficits such as motor incoordination.

The beam-walk apparatus has been used to assess sensorimotor deficits following brain injury and other conditions, resulting in altered gait, balance and/or proprioception.[23] The beam-walk apparatus was found to be a sensitive measure for the evaluation of diabetes-induced sensorimotor changes.[9] In this method, the number of hind paw slips as mice crossed the beam-walk apparatus was used to access the sensorimotor ability. For a slip to be counted, the foot had to lose during walking on the beam-walk apparatus. After 10 weeks of hyperglycemia, the diabetic mice showed a significantly greater number of foot slips than the nondiabetic mice, which is a marker of diabetes-induced large fiber damage.[9]

## Functional biomarkers

Functional biomarkers include nerve conduction slowing and resistance to ischemic conduction block in large fibers, slowing nerve blood flow, delayed gastric emptying and intestinal and colonic transit.

**a) Slow sciatic nerve conduction velocity (SNCV) and motor nerve conduction velocity:** Electrophysiological measures of nerve impairment are the “gold standard” for determining sensory and motor nerve function, which include assessment of motor and sensory nerve conduction in the tail and sciatic nerve. A NCV test is an electrical test that is used to determine the adequacy of the conduction of the nerve impulse as it courses down a nerve. This test is used to detect signs of nerve injury.

Diabetic rodents manifest slow SNCV and motor NCV. Growing evidence indicates that the etiology of diabetes-induced nerve dysfunction is far more complex and involves both vascular and non-vascular mechanisms.[4] NCV decrease clearly in diabetic rodents from the second week to the eighth week.[5,6,24] Early symptoms of reduced NCV are observed within the second week of diabetes, with worsening of the symptoms from the second week to the eighth week in diabetic rodents.

**b) Reduced nerve blood flow (NBF):** Reduction in NBF was reported in diabetic rats after 8 weeks of diabetes induction.[5] Decreased NBF with resulting hypoxia is one of the mechanisms responsible for reduced NCV in diabetic conditions.[24] Several studies have reported a causal relation between peripheral nerve perfusion and conduction deficits.[25,26] In addition to this, nitrosative stress has been reported to contribute to conduction deficits seen in an animal model of type 2 diabetes.[27] Impaired endothelium-dependent vasodilatation may be among the causes of reduced NBF in diabetic animals. There are also reports indicating that poly (ADP-ribose) polymerase (PARP) overactivation contributes to endothelial dysfunction, which might be responsible for NBF deficits.[28] Various techniques reported for the assessment of nerve blood flow include H2-polarographic technique,[29] ^14^C-labelled iodoantipyrine technique[30] and laser Doppler velocimetry.[31] Each technique has its advantages and disadvantages. The more appropriate technique out of these is the H2-polarographic technique to measure NBF because of its ability to separate nutritive (capillary) from capacitative (arteriovenous) flow and its ability to measure NBF repeatedly under various physiological conditions.

**c) Delayed gastric emptying, intestinal and colonic transit:** Many gastrointestinal symptoms may accompany diabetic autonomic neuropathy (DAN), and the most common among them is gastroparesis. Delayed gastric emptying (gastroparesis) was reported to occur in approximately 50% of the patients with longstanding diabetes.[32] Markers of gastroparesis reported in diabetic rodents include delayed gastric emptying, intestinal and colonic transit.[33,34] This may be due to damage of the peripheral cholinergic neurons.[33] Intestinal transit of charcoal meal can be determined by modified Janseen method. Delayed gastric emptying, small intestinal and colonic transit of the phenol red meal was reported in ALX-induced diabetes after 4 weeks.[34] Bijender *et al*.[33] reported a decrease in the small intestinal transit of charcoal meal in STZ-induced diabetes after 8 weeks.

## Structural biomarkers

Structural biomarkers of neuropathy in diabetic rodents include epidermal fiber numbers/morphology in small fibers and axonal number/caliber in nerve trunks of large fibers.

**a) Loss of epidermal nerve fiber:** Epidermal nerve fiber quantification through skin biopsies are emerging as a valuable means of diagnosing as well as staging peripheral nerve disorders. It is a minimally invasive technique and it allows for the assessment of a variety of fiber types, including the small unmyelinated fibers that are difficult to evaluate by other means. This technique provides a means for the assessment of DN, both to stage and to evaluate the progression of neuropathy as well as to assess the efficacy of potential therapeutics. Epidermal nerve fibers are capsaicin-sensitive unmyelinated C-fibers involved in detecting thermal nociceptive pain.

One of the studies assessed substance P (SP), calcitonin gene-related peptide (CGRP), vasoactive intestinal polypeptide (VIP), neuropeptide Y (NPY) and protein gene product 9.5 (PGP9.5) immunoreactivity in skin from the lip and footpad of rats injected with STZ. At 12 weeks of diabetes, an increase in the CGRP-reactive fibers was observed in both the dermis and the epidermis, along with an increase in VIP around the sweat glands and blood vessels.[35] There is also a report of an increase in PGP9.5 immunoreactivity, while dermal or epidermal SP and NPY immunoreactivity was unchanged.[35] In a study examining the cutaneous innervation of insulin-deficient C57Bl/6 mice with STZ-induced diabetes of 7 weeks duration, the footpad and flank skin biopsies showed decreased immunoreactivity to CGRP, P2X3 and PGP9.5 when compared to nondiabetic controls.[36] In yet another study using STZ-diabetic thy1-YFP mice, which express a yellow fluorescent protein (YFP) in their nerve fibers that allows for noninvasive monitoring of cutaneous innervation, a reduction was observed after 3 months of diabetes.[36] There is also a report of reductions in PGP9.5-immunoreactive intraepidermal nerve fiber (IENF) densities in both C57Bl/6 and Swiss Webster mice as early as 4 weeks after the induction of diabetes with STZ.[37] Studies have also been performed in a type 2 diabetic mouse model that spontaneously develops insulin resistance before ultimately progressing to insulin deficiency.[38] Compared to their heterozygous nondiabetic littermates, PGP9.5-immunoreactive epidermal fibers were significantly reduced in genetically diabetic C57BL/KsJ-m+/+Leprdb db/db mice, and similar reductions were observed in the skin samples from human subjects evaluated in the same study.[39] In C57Bl6/J ob/ob mice, another model of type 2 diabetes, a decrease in the IENF density was observed in animals that were approximately 11 weeks old.[27]

Diabetes-induced changes in the IENF density in rats and mice suggest that measurement of the IENF density provide a valuable biomarker for the assessment of diabetic neuropathies, investigating etiologic mechanisms and the evaluation of therapeutic efficacy.

**b) High variability in axonal width:** Muscle spindle quantification has involved cross-sections of the spindle capsule through light or electron microscopy and to calculate the mean width of three or more axonal rotation and mean interrotational distance (IRD), i.e. the space in between the axonal rotations. This method of visualizing the spindle does not allow for intricate analysis of axon morphology. Visualizing muscle spindle afferent longitudinally provides more information about axonal width and IRD. A study in STZ-induced c57BL/6 diabetic mice suggests that diabetic muscle spindle Ia fibers have a high variability in their axonal width and IRD as a group and within individual muscle spindle.[9] This technique now provides a new means to determine the efficacy of therapeutic interventions aimed at improving large-fiber DN. Because it is difficult to access spindle innervations in human muscle biopsies, it reinforces the need for animal model studies to address the pathogenesis and treatment of human large-fiber DPN.

**c) Reduced axonal number and caliber:** It gives information about the morphological changes that occur due to diabetes in large fibers. Diabetic mice reported to have smaller mean fiber, thinner myelin in tibial fascicles, preserved fiber density and numbers but no changes were observed in axonal diameter of sural nerves populated by sensory (and some autonomic) axons.[40]

## Molecular biomarkers

Molecular biomarkers of DN include down-regulation of structural proteins, e.g. neurofilament, tubulins and growth factor, and up-regulation of markers of neurotoxicity, such as nitrotyrosine, rise in intracellular calcium and overactivation of poly (ADP-ribose) polymerase. Activated caspase, receptor for advanced glycation end products (RAGE) and nuclear factor kappa B (NFkB) increase polyol, including nerve sorbitol and fructose content, which was also manifested in diabetic rodents.

In addition to these, other molecular biomarkers of neuropathy reported in diabetic rodents are as follows:

**a) Down regulation of structural protein and nerve growth factor (NGF):** The goal in treating DN is not only to prevent the progression of neuropathic symptoms and nerve dysfunction and degeneration but also to promote regeneration of degenerated nerve fibers. Because the recovery of diseased nerve function depends on both regeneration of degenerated nerve fibers and reestablishment of their functional connections with the target tissue, it is important to establish animal models in order to examine how diabetes influences nerve regeneration and whether potential therapeutic agents can promote satisfactory regeneration of functioning nerve fibers. NGF and structural proteins are required not only for the development and regeneration but also for the maintenance of sensory and autonomic neurons and their axons, which serve as biomarkers of neuropathy in animal models of diabetes. Most of their serum and nerve levels have been reported to be decreased in the diabetic condition. The decreased nerve regenerative capacity in diabetes has been associated with impaired neurotrophic tone, which could reflect diminished synthesis, secretion or responsiveness of neurotrophic factors such as NGF and structural protein in sensory and autonomic nerve fibers. Compromised neuronal function, atrophy of neurons or nerves and even neuronal death may be induced due to growth factor reduction in DN. Various factors of nerve regeneration that are reported to down-regulate in experimental neuropathy in diabetic rodents were reviewed by Yasuda *et al*.,[41] and are enlisted in Table 5.

**Table 5:** Factors of nerve regeneration that are reported to down-regulate in experimental neuropathy in diabetic rodents

*Factors of nerve regeneration*	*Location where concentration is down-regulated*
Growth-associated protein-43 (GAP-43)	Dorsal root of ganglia (DRG)
Tα1α-tubulin	DRG
Nerve growth factor (NGF)	Serum, nerve
Brain-derived neurotrophic factor (BDNF)	Nerve, DRG
Neurotrophin-3	Muscle, nerve
Neurotrophin-4/5	Nerve
Insulin-like growth factor-I (IGF-I)	Superior cervical ganglia
Insulin-like growth factor-II (IGF-II)	Nerve, spinal cord, brain
Insulin-like growth factor-binding protein (IGFBP-3)	Serum

**b) Down-regulation of receptors of neurotrophins:** Neurotrophins bind to two classes of receptors: tyrosine receptor kinases or trks (trk A, trk B, trk C) and a low-affinity receptor, “p75.” It was reported that the expression of p75NTR was decreased in DRG of STZ-induced diabetic rats compared with those of control rats,[42] whereas that of trk A was unchanged.[42] No increase in plasma p75NTR IR was observed after 4 weeks of diabetes, but significant increases were present at 10 and 12 weeks of diabetes. The beginning of this trend was visible at 8 weeks after the induction of diabetes.[43]

**c) Biomarkers of oxidative stress:** Rats subjected to chronic STZ-induced diabetes showed a significant increase in brain malondialdehyde (MDA) and nitric oxide (NO) levels as biomarkers of oxidative stress and also a significant decrease in the antioxidant systems, glutathione (GSH), glutathione reductase (GR), glutathione peroxidase (GPX), glutathione S-transferase (GST) and superoxide dismutase (SOD) activities.[44]

In addition to this, other biomarkers at the molecular level reported in diabetic rodents include activation of enzyme such as aldose reductase, poly(ADP-ribose) polymerase, activated caspase, RAGE, increased nerve content of glucose, sorbitol and fructose, reduced nerve myoinositol, taurine and lower (Na/K)^+^-ATPase activity.

## Conclusion

Neuropathy is a common complication of diabetes and multiple pathological factors are responsible for the development of neuropathy. A number of animal models of diabetes develop neuropathy, but in different ways, depending on the background strain, diet composition, insulin/C-peptide deficiency, coexisting hyperglycemia and hypertension and duration of diabetes. Although rodent models of DN do not fully replicate the pathology observed in human patients, yet they are commonly used for the evaluation of drugs that cannot be administrated to humans unless previously tested in animals. The differences may explain problematic results with various forms of treatment that appear successful in animal models but show disappointing results in human trials. Therefore, care must be taken while extrapolating results of animal studies to that of human studies.

To evaluate the novel drug with potential activity in DN, it is essential to select an animal model and biomarkers of neuropathy that will most accurately predict a clinical response. This review will provide useful knowledge of biomarkers of neuropathy in both type 1 and type 2 diabetic rodents, which may allow us to predict the early onset of complications and identify the new possible treatments.
